# Morphologic evidence of telocytes in human thyroid stromal tissue

**DOI:** 10.1111/jcmm.17282

**Published:** 2022-03-20

**Authors:** Irene Rosa, Lidia Ibba‐Manneschi, Daniele Guasti, Giuliano Perigli, Maria‐Simonetta Faussone‐Pellegrini, Mirko Manetti

**Affiliations:** ^1^ 9300 Section of Anatomy and Histology Department of Experimental and Clinical Medicine University of Florence Florence Italy; ^2^ 9300 Unit of Endocrine Surgery Department of Experimental and Clinical Medicine University of Florence Florence Italy

**Keywords:** CD34, human thyroid, PDGFRα, stromal cells, telocytes

## Abstract

Despite the evidence accumulated over the past decade that telocytes (TCs) are a distinctive, though long neglected, cell entity of the stromal microenvironment of several organs of the human body, to date their localization in the endocrine glands remains almost unexplored. This study was therefore undertaken to examine the presence and characteristics of TCs in normal human thyroid stromal tissue through an integrated morphologic approach featuring light microscopy and ultrastructural analysis. TCs were first identified by immunohistochemistry that revealed the existence of an intricate network of CD34^+^ stromal cells spread throughout the thyroid interfollicular connective tissue. Double immunofluorescence allowed to clearly differentiate CD34^+^ stromal cells lacking CD31 immunoreactivity from neighbour CD31^+^ microvascular structures, and the evidence that these stromal cells coexpressed CD34 and platelet‐derived growth factor receptor α further strengthened their identification as TCs. Transmission electron microscopy confirmed the presence of stromal cells ultrastructurally identifiable as TCs projecting their characteristic cytoplasmic processes (i.e., telopodes) into the narrow interstitium between thyroid follicles and blood microvessels, where telopodes intimately surrounded the basement membrane of thyrocytes. Collectively, these morphologic findings provide the first comprehensive demonstration that TCs are main constituents of the human thyroid stroma and lay the necessary groundwork for further in‐depth studies aimed at clarifying their putative implications in glandular homeostasis and pathophysiology.

## 1 INTRODUCTION

During the past decade, we witnessed a substantial reappraisal of the microscopic anatomy of a variety of human organs based on the new evidence that telocytes (TCs) are interstitial/stromal cells with specific ultrastructural features, making them distinguishable from other fibroblast‐like cells.^1^, ^2^, ^3^, ^4^ Indeed, TCs feature very long and slim cytoplasmic extensions (‘telopodes’) with a characteristic varicose/moniliform shape conferred by the alternation of extremely thin segments (‘podomers’) and small cistern‐like dilated portions (‘podoms’), which are arranged in networks that delimit parenchymal cell structures, surround microvessels and nerves, and contact other cell types of the stromal microenvironment.^1^, ^2^, ^3^, ^4^ Whether TCs present distinctive cluster of differentiation (CD) antigens is still unknown, but currently they are identified mainly on the basis of their CD34^+^ immunophenotype (i.e., TCs/CD34^+^ stromal cells) or the coexpression of CD34 and platelet‐derived growth factor receptor α (PDGFRα or CD140a).^2^, ^4^, ^5^, ^6^, ^7^, ^8^ Putative functions of TCs include the regulation of tissue morphogenesis and homeostasis through both direct intercellular contacts and transfer of extracellular vesicles carrying molecular signals to neighbouring cells, as well as their participation to a number of either tissue regenerative or pathophysiologic processes, among others.^2^, ^3^, ^4^, ^5^, ^7^, ^9^, ^10^, ^11^

In humans, to date TCs have been widely studied in organs of the cardiocirculatory, respiratory, urogenital and digestive systems, including both the gastrointestinal tract and exocrine digestive glands, whereas their localization in the endocrine glands remains almost unexplored.^3^, ^4^ Therefore, we undertook the present research to gain insights into the presence and characteristics of TCs in the human thyroid gland.

## 2 MATERIALS AND METHODS

### 2.1 Tissue samples

The study employed paraffin‐ and epoxy resin‐embedded normal human thyroid tissue samples from the archives of the Section of Anatomy and Histology, Department of Experimental and Clinical Medicine, University of Florence. Specimens without any obvious parenchymal lesion had been collected from five adult individuals after thyroid surgery with the approval of the Institutional Review Board at the Careggi University Hospital, Florence, Italy. All individuals had signed an informed consent form in accordance with the Declaration of Helsinki.

### 2.2 Light microscopy

Formalin‐fixed, paraffin‐embedded tissue sections (5 μm thick) were deparaffinized and subjected to either routine haematoxylin and eosin (H&E) staining or heat‐induced antigen retrieval in sodium citrate buffer (10 mM, pH 6.0) followed by immunohistochemistry or immunofluorescence staining according to highly standardized and validated protocols.^6^, ^8^, ^12^ Tissue sections were incubated in 3% H_2_O_2_ solution for 15 min at room temperature to block endogenous peroxidase activity and subsequently subjected to immunoperoxidase‐based immunohistochemistry by using the UltraVision Detection System (Anti‐Polyvalent, HRP, catalog number TP‐125‐HL; Lab Vision) and a mouse monoclonal anti‐human CD34 antibody (1:50 dilution; overnight incubation at 4°C; catalog number M7165; Dako) as described in detail elsewhere.^6^ The immunoreaction was developed with 3‐amino‐9‐ethylcarbazole (AEC kit, catalog number TA‐125‐SA; Lab Vision) as chromogenic substrate, followed by nuclear counterstain with haematoxylin.

For immunofluorescence staining, after deparaffinization and exposure to a glycine solution (2 mg/ml) for 10 min to quench autofluorescence, tissue sections (5 μm thick) were blocked for 1 h at room temperature with 1% bovine serum albumin in phosphate‐buffered saline. A mixture of mouse monoclonal anti‐human CD34 (1:50 dilution; catalog number M7165; Dako) and rabbit polyclonal anti‐human CD31 (1:50 dilution; catalog number ab28364; Abcam) or goat polyclonal anti‐human PDGFRα (1:100 dilution; catalog number AF‐307‐NA; R&D Systems) antibodies was applied to tissue sections overnight at 4°C. After extensive washing, tissue slides were incubated with a mixture of Alexa Fluor‐488‐conjugated donkey anti‐mouse and Rhodamine Red‐X‐conjugated goat anti‐rabbit IgG or Alexa Fluor‐568‐conjugated donkey anti‐mouse and Alexa Fluor‐488‐conjugated donkey anti‐goat IgG (1:200 dilution; Invitrogen) for 45 min at room temperature in the dark. Nuclei were counterstained with 4′,6‐diamidino‐2‐phenylindole (DAPI). Negative controls were performed using irrelevant isotype‐ and concentration‐matched mouse, rabbit and goat IgG. The immunostained sections were examined under a Leica DM4000 B microscope and photographed with a Leica DFC310 FX 1.4‐megapixel digital colour camera equipped with the Leica software application suite LAS V3.8 (Leica Microsystems).

### 2.3 Transmission electron microscopy (TEM)

Transmission electron microscopy was performed as detailed elsewhere.^6^, ^12^ Small tissue fragments were fixed with 4% cacodylate‐buffered glutaraldehyde (pH 7.4), post‐fixed in 1% OsO_4_ and embedded in Epon 812 resin (Sigma‐Aldrich). Tissue blocks were cut with an RMC MT‐X ultramicrotome (EMME3). Ultrathin sections (~70 nm thick) were stained with UranyLess (Electron Microscopy Sciences) and alkaline bismuth subnitrate solutions, followed by examination under a JEOL JEM‐1010 electron microscope equipped with a MegaView III high‐resolution digital camera and imaging software (Jeol).

## 3 RESULTS

### 3.1 Immunohistochemical identification of TCs in the human thyroid stroma

**FIGURE 1:**
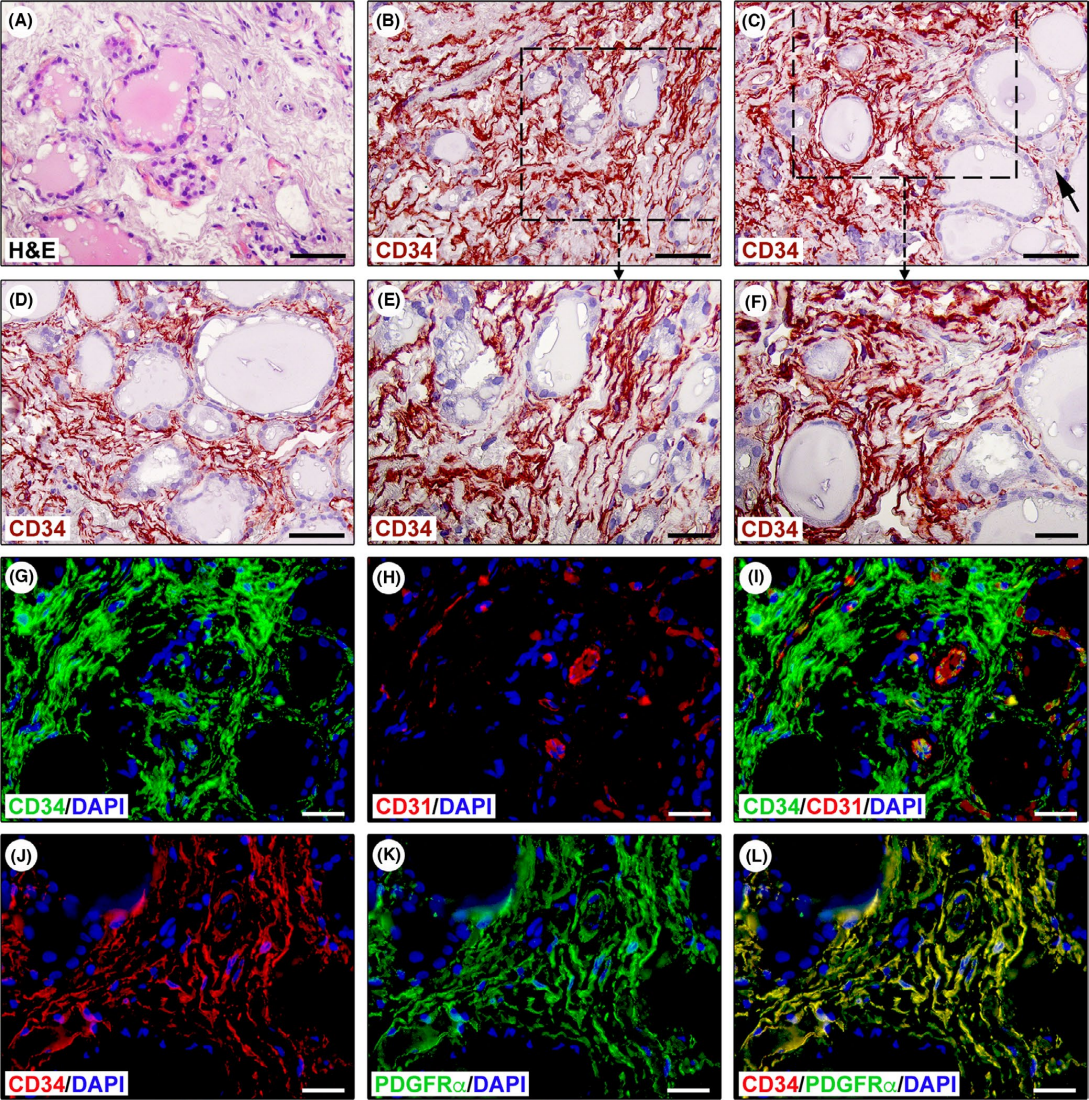
Immunohistochemical identification of telocytes (TCs)/CD34^+^ stromal cells in normal human thyroid tissue sections. (A) Haematoxylin and eosin (H&E) staining testifying the normal appearance of thyroid tissue consisting of colloid‐containing follicles lined with epithelial cells (thyrocytes) and interfollicular reticular connective tissue harbouring fibroblasts, mononuclear cells, nerve fibres and small blood vessels and lymphatics. (B–F) CD34 immunohistochemistry with haematoxylin nuclear counterstain showing the presence of an intricate network of CD34^+^ stromal cells spread throughout the interfollicular connective tissue. Representative photomicrographs of tissue sections from three different specimens are shown. (E and F) Higher magnifications of the boxed areas in (B and C). CD34^+^ stromal cells exhibit the typical TC morphology, that is spindle‐shaped cells with very long cytoplasmic processes displaying an irregular calibre and a sinuous trajectory (E and F). CD34^+^ TCs are numerous around microfollicles, while they are less represented around macrofollicles (C, arrow). (G–I) Double immunofluorescence staining for CD34 (green) and pan‐endothelial marker CD31 (red) with DAPI (blue) counterstain for nuclei. The interfollicular network of TCs/CD34^+^ stromal cells lacks CD31 immunoreactivity and surrounds CD31^+^ microvessels (G–I). (J–L) Double immunofluorescence staining for CD34 (red) and platelet‐derived growth factor receptor α (PDGFRα, green) with DAPI (blue) counterstain for nuclei. All TCs/CD34^+^ stromal cells located in the interfollicular connective tissue display PDGFRα immunoreactivity (J–L). Scale bar: 50 μm (A–D), 25 μm (E–L)

The analysis of haematoxylin and eosin‐stained thyroid sections allowed both to exclude any obvious parenchymal alterations and to identify tissue specimens with well‐preserved stroma suitable for subsequent immunohistochemical identification of TCs (Figure 1A).

As shown in Figure 1B–F, an intricate network of CD34^+^ cells displaying the characteristic TC morphology (i.e., TCs/CD34^+^ stromal cells) was widespread in the interfollicular stromal compartment. Indeed, these interstitial cells were spindle‐shaped and had very long and varicose cytoplasmic processes that often exhibited a sinuous trajectory (Figure 1E,F). Though numerous CD34^+^ TCs were clearly identifiable around microfollicles, they appeared less represented in the narrow stroma between macrofollicles (Figure 1C). Since CD34 expression can be found also in the endothelium of blood microvessels that form an extensive bed around thyroid follicles, we additionally performed double immunofluorescence for CD34 and the pan‐endothelial marker CD31 (Figure 1G–I). This analysis confirmed the presence of a diffuse interstitial network of CD31^−^/CD34^+^ TCs surrounding both thyroid follicles and CD31^+^ microvessels (Figure 1G–I). Furthermore, all TCs spread throughout the thyroid interfollicular connective tissue were CD34^+^/PDGFRα^+^ (Figure 1J–L).

### 3.2 Ultrastructural identification of TCs in the human thyroid stroma

**FIGURE 2:**
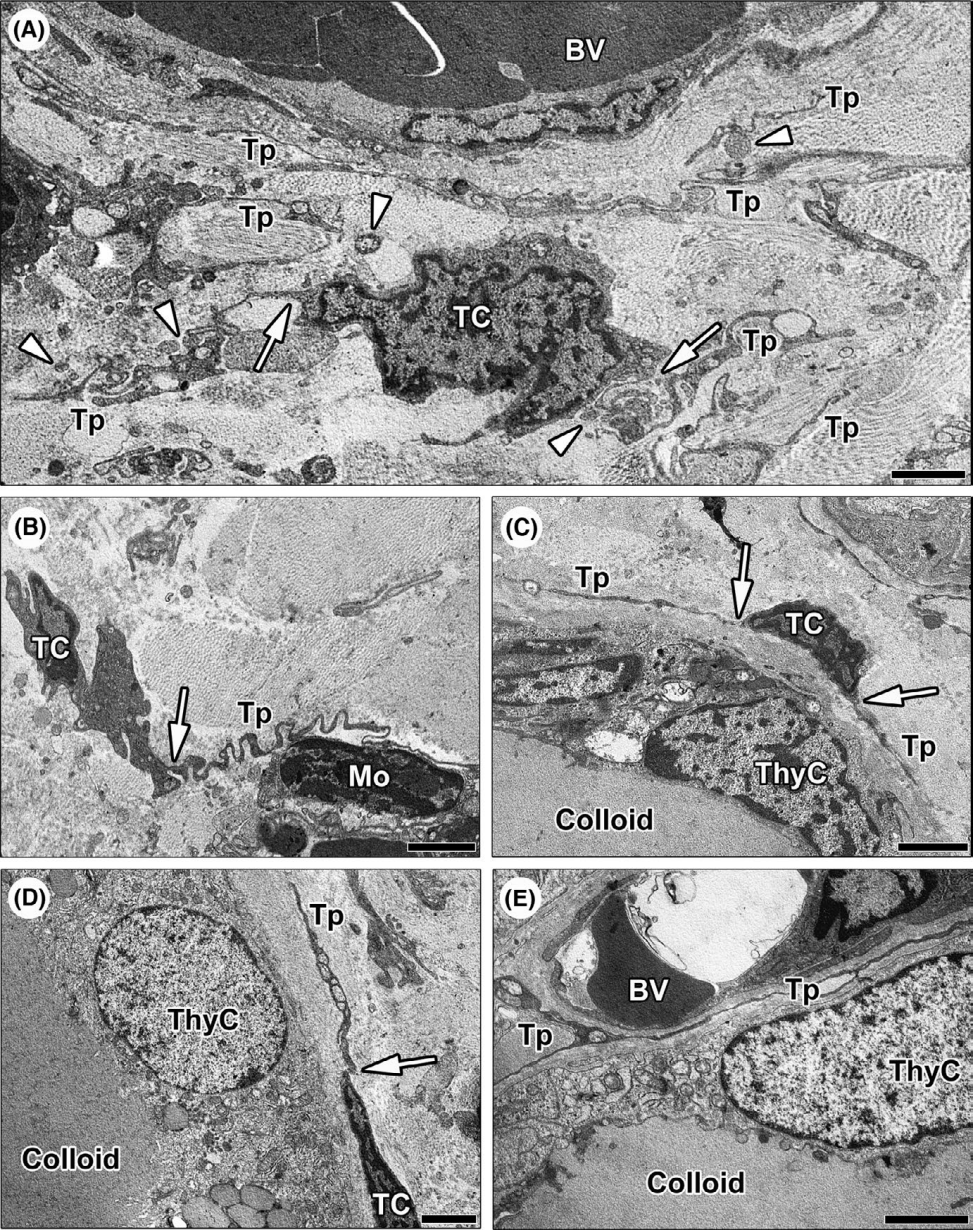
Ultrastructural identification of telocytes (TCs) in normal human thyroid stromal tissue. (A–E) Representative transmission electron microscopy photomicrographs of thyroid ultrathin sections stained with UranyLess and bismuth subnitrate solutions. (A–D) TCs are ultrastructurally identifiable as stromal cells with long cytoplasmic projections (telopodes) characterized by a narrow emergence from the cell body (arrows) and a moniliform profile due to the alternation of thin segments (podomers) and expanded portions (podoms); the cell body of TCs is spindle‐shaped, oval, or piriform and mostly occupied by the nucleus. (A) Note a bipolar TC giving rise to two convoluted telopodes; the labyrinth‐like network formed by telopodes extends in between collagen bundles and around blood microvessels. Numerous extracellular vesicles are present nearby telopodes (arrowheads). (B) A TC projects a telopode with a very sinuous trajectory to intimately encircle and contact a mononuclear cell. (C and D) The telopodes of TCs are arranged around the basement membrane of thyrocytes lining colloid‐containing follicles. (E) Telopodes extend into the narrow interstitium between thyroid follicles and blood microvessels. Scale bar: 2 μm (A–E). BV, blood vessel; Mo, mononuclear cell; TC, telocyte; ThyC, thyrocyte; Tp, telopode

Transmission electron microscopy confirmed the existence of stromal cells ultrastructurally identifiable as TCs with the same tissue distribution revealed by CD34/PDGFRα immunostaining (Figure 2A–E). The ultrastructural hallmark of TCs located in the thyroid stroma was that they presented with very long, slender and moniliform telopodes with a narrow emergence from the cell body, which was spindle‐shaped, oval, or piriform and contained a large nucleus surrounded by a scarce cytoplasm (Figure 2A–D). Telopodes appeared often convoluted and formed a labyrinth‐like network that extended in between collagen bundles and around blood microvessels, and established close relationships with other cell types, such as mononuclear cells (Figure 2A,B). Moreover, extracellular vesicles were frequently detected nearby telopodes (Figure 2A). As displayed in Figure 2C–E, the telopodes of TCs were intimately arranged around the basement membrane of thyrocytes lining colloid‐containing follicles and extended into the narrow stromal space between follicles and blood microvessels.

## 4 DISCUSSION

Although CD34^+^ stromal fibroblastic or dendritic cells were long noticed in the thyroid stroma,^5^, ^13^, ^14^ here it is conclusively proven for the first time that such stromal cells are, indeed, TCs. In fact, we discovered that the main constituent of the human thyroid stromal tissue is a reticular network of cells displaying the typical ultrastructural and CD34^+^/PDGFRα^+^ immunophenotypic features of TCs. These TCs appear to be highly organized with their telopodes that closely wrap the abluminal side of thyroid follicles separating them form the surrounding capillaries. In keeping with the diverse roles ascribed to the TCs in other organs,^1^, ^2^, ^3^, ^4^, ^5^, ^9^ such an interfollicular network of TCs could uniquely contribute to thyroid tissue homeostasis and plasticity by providing mechanical support and molecular signals to thyroid follicles which continuously change their size and shape depending on the thyrotropin‐regulated colloid content. Indeed, the TC network seems to faithfully adapt to such thyroid follicle changes, as testified by the evidence that TCs are more represented around microfollicles than around macrofollicles. Moreover, by strategically interposing between thyroid follicles and blood capillaries, the network of telopodes might be involved in the regulation of thyroid hormone release. Of note, a previous study identified stromal cells coexpressing CD34 and thyrotropin receptor in thyroid tissue from donors with Graves’ disease, Hashimoto's thyroiditis, as well as in normal‐appearing thyroid tissue.^15^ Taken together, it is conceivable that thyroid follicles and interfollicular TCs constitute a single, thyrotropin‐driven functional unit. In addition, it is tempting to speculate that TCs might participate in the pathogenesis of thyroid autoimmunity after thyrotropin receptor activation, thus representing novel potential therapeutic targets.

To conclude, we are confident that our findings pave the way for further studies that will hopefully elucidate the many putative functional implications of TCs in thyroid gland pathophysiology.

## CONFLICT OF INTEREST

The authors confirm that there are no conflicts of interest.

## AUTHOR CONTRIBUTIONS

**Irene Rosa:** Data curation (equal); Formal analysis (equal); Investigation (equal); Methodology (equal); Validation (equal); Writing – review & editing (equal). **Lidia Ibba‐Manneschi:** Data curation (equal); Formal analysis (equal); Investigation (equal); Validation (equal); Writing – original draft (equal); Writing – review & editing (equal). **Daniele Guasti:** Investigation (equal); Methodology (equal); Writing – review & editing (equal). **Giuliano Perigli:** Formal analysis (equal); Investigation (equal); Writing – review & editing (equal). **Maria‐Simonetta Faussone‐Pellegrini:** Formal analysis (equal); Investigation (equal); Writing – original draft (equal); Writing – review & editing (equal). **Mirko Manetti:** Conceptualization (lead); Data curation (equal); Formal analysis (equal); Funding acquisition (lead); Investigation (equal); Methodology (equal); Supervision (lead); Validation (equal); Writing – original draft (lead); Writing – review & editing (equal).
